# Where is that smell coming from?

**DOI:** 10.7554/eLife.82635

**Published:** 2022-09-20

**Authors:** Samuel Brudner, Thierry Emonet

**Affiliations:** 1 Department of Molecular, Cellular and Developmental Biology, and the Quantitative Biology Institute, Yale University New Haven United States; 2 Department of Molecular, Cellular and Developmental Biology, the Quantitative Biology Institute, and the Department of Physics, Yale University New Haven United States

**Keywords:** olfactory navigation, turbulence, decision-making, foraging, None

## Abstract

Computational model reveals why pausing to sniff the air helps animals track a scent when they are far away from the source.

**Related research article** Rigolli N, Reddy G, Seminara A, Vergassola M. 2022. Alternation emerges as a multi-modal strategy for turbulent odor navigation. *eLife* **11**:e76989. doi: 10.7554/eLife.76989.

Dogs, rodents and many other animals with a strong sense of smell often track a scent by keeping their nose to the ground, occasionally pausing to raise their heads and sniff something mysterious in the air (Jinn et al., 2020; Gire et al., 2016). However, exactly how alternating between these two behaviors helps animals navigate to the source of an odor remains unclear.

Airborne odors are transported by the wind, making them subject to the twisting and stretching of turbulent air motions. This results in animals downwind from an odor source being more likely to smell the odor intermittently, as air pockets containing the scent are interspersed with periods of clean air (Celani et al., 2014; Connor et al., 2018). Studies in insects suggest that animals surge upwind when they detect a smell in order to keep in contact with these turbulent odor plumes; when no odor is detected, they cast crosswind instead (Álvarez-Salvado et al., 2018; Demir et al., 2020; Kennedy, 1983; Flügge, 1934). Now, in eLife, Nicola Rigolli, Gautam Reddy, Agnese Seminara and Massimo Vergassola report how pausing to sniff the air when casting crosswind helps animals navigate towards the source of an odor (Rigolli et al., 2022).

To investigate how alternating behaviors impacts odor navigation, the team (who are based at institutes in France, Italy and the United States) designed a virtual search environment by simulating an odor dispersing downwind over a large area. The set-up created a challenging search scenario, including a low probability of encountering an odor pocket far from the source. Using machine learning, computer programs trained ‘artificial navigating agents’ to find the origin of the smell as quickly as possible (Sutton and Barto, 2019). During their search, these artificial agents were allowed to alternate between ‘walking’ while sniffing close to the ground and stopping to smell the air. Information gathered from these behaviors allowed agents to decide where to go next. After each attempt, the agents could use feedback about their previous search times to modify their strategy in the next trial. Although the researchers did not impose any explicit strategy or solution, agents reliably learnt that stopping to sniff the air sped up their search, even though it required them to pause.

Notably, trained agents mostly stopped to smell the air during the initial phase of their search before they had detected any odor. This suggests that alternating between ground and air sniffing helps agents to explore areas with dispersed levels of odor more efficiently (Figure 1A). Once agents successfully encounter an airborne cue, this signals that they have entered an odor rich zone and their rate of alternation drastically decreases.

**Figure 1.**
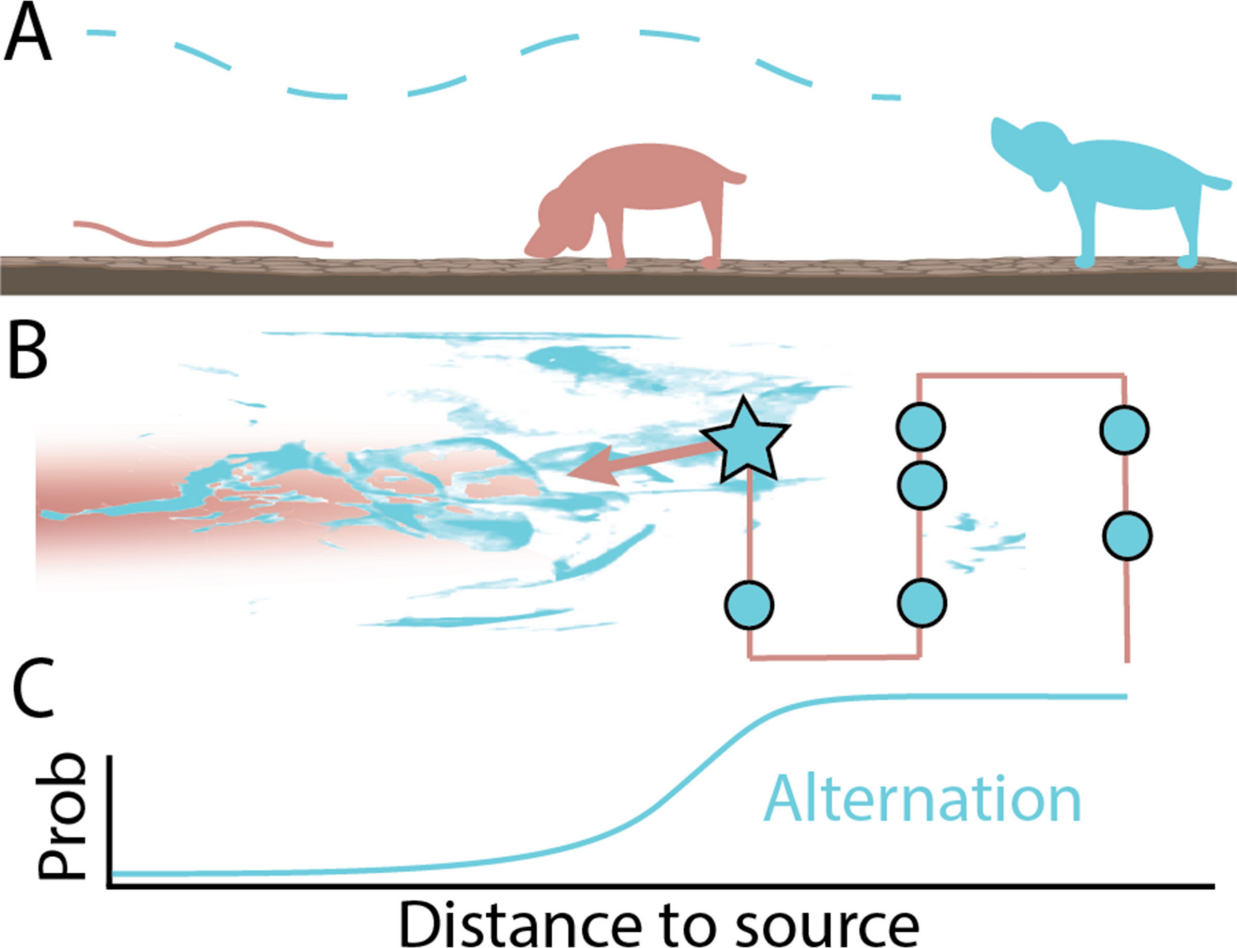
The optimal strategy for finding the source of a smell. (**A**) When tracking the source of an odor, animals alternate between walking while sniffing the ground (brown) and pausing to sniff the air (blue). Animals sniff the air more frequently when they are further away from the source and airborne cues are more dispersed (blue dashed line). As they get nearer and the density of the airborne cues increases (brown line), animals alternate less frequently and track the scent by sniffing close to the ground. (**B**) Rigolli et al. used a machine learning algorithm to identify the optimal strategy for tracking odors in the wind. They simulated an odor dispersing in the air (blue plume) and close to the ground (brown plume) and then trained artificial agents to find the source of the smell: the brown line indicates the trajectory agents took whilst sniffing the floor, and the blue circles represent where they paused to sniff the air. The algorithm revealed that the best way for agents to find the source of the odor was for them to alternate to sniffing the air when moving crosswind, and intersperse this with occasional surges forward until an odor was detected (blue star). (**C**) The simulation showed that agents displayed this alternating behavior less frequently as they moved closer to the odor source.

Image credit: Samuel Brudner; odor plumes in panel B are based on images by Nirag Kadakia and Mahmut Demir.

But how exactly does alternation speed up getting that first hint of a scent? Failing to detect an odor in a region indicates that the source of the smell is unlikely to be upwind of this area, eliminating the need to search there in the future. Odors disperse slower near the ground, and as a result do not reach as far as odors travelling in the air. Sniffing above their heads therefore allows agents to rule out larger upwind areas (if no smell is present), while also increasing the likelihood of detecting faint signals that are absent at ground level.

The simulation also revealed that during the early search phase, trained agents combined alternation with specific patterns of locomotion (Figure 1B). Agents moving crosswind sniffed the air more frequently than when they surged upwind. Rigolli et al. observed that this behavior helps the agents to rule out cross-sections of the simulated arena before moving upwind to gather evidence about a new region.

This cast-sniff-surge strategy involves many tradeoffs: casting over a wider distance takes longer but also eliminates a wider cross-section; sniffing in the air requires stopping and therefore losing time. Using a mathematical framework, Rigolli et al. show that optimally balancing these tradeoffs requires exploring the arena back and forth in an expanding crosswind zigzag, gradually casting across larger areas as the search progresses. Remarkably, these characteristics also appeared in trained agents which were not constrained to use a cast-sniff-surge approach.

Overall, Rigolli et al. demonstrate how, in theory, alternating between sniffing the ground and the air allows animals to efficiently search large areas for an odor source. Future studies should now test these predictions, for example examining if real animals do tend to alternate behaviors mostly in odor-poor regions. This work could also be applied to robotics, in particular to improve the exploratory behavior of drones used in difficult search-and-rescue operations.
